# Tobacco Product Use Among Adults – United States, 2021

**DOI:** 10.15585/mmwr.mm7218a1

**Published:** 2023-05-05

**Authors:** Monica E. Cornelius, Caitlin G. Loretan, Ahmed Jamal, Brittny C. Davis Lynn, Margaret Mayer, Iris C. Alcantara, Linda Neff

**Affiliations:** ^1^Office on Smoking and Health, National Center for Chronic Disease Prevention and Health Promotion, CDC; ^2^Office of Science, Center for Tobacco Products, U.S. Food and Drug Administration, Silver Spring, Maryland; ^3^Tobacco Control Research Branch, National Cancer Institute, National Institutes of Health, Washington, DC.

Commercial cigarette smoking among U.S. adults has declined during the preceding 5 decades (*1*,*2*); however, tobacco product use remains the leading cause of preventable disease and death in the United States, and some populations continue to be disproportionately affected by tobacco use (*1*,*2*). To assess recent national estimates of commercial tobacco use among U.S. persons aged ≥18 years, CDC, the Food and Drug Administration (FDA), and the National Cancer Institute analyzed 2021 National Health Interview Survey (NHIS) data. In 2021, an estimated 46 million U.S. adults (18.7%) reported currently using any tobacco product, including cigarettes (11.5%), e-cigarettes (4.5%), cigars (3.5%), smokeless tobacco (2.1%), and pipes (including hookah)* (0.9%).^†^ Among those who used tobacco products, 77.5% reported using combustible products (cigarettes, cigars, or pipes), and 18.1% reported using two or more tobacco products.^§^ The prevalence of current use of any tobacco product use was higher among the following groups: men; persons aged <65 years; persons of non-Hispanic other races; non-Hispanic White (White) persons^¶^; residents of rural (nonmetropolitan) areas; financially disadvantaged (income-to-poverty ratio = 0–1.99); lesbian, gay, or bisexual (LGB) persons; those uninsured or enrolled in Medicaid; adults whose highest level of education was a general educational development (GED) certificate; who had a disability; and who had serious psychological distress. Continued surveillance of tobacco product use, implementation of evidence-based tobacco control strategies (e.g., hard-hitting media campaigns, smoke-free policies, and tobacco price increases), conducting linguistically and culturally appropriate educational campaigns, and FDA regulation of tobacco products will aid in reducing tobacco-related disease, death, and disparities among U.S. adults (*3*,*4*).

NHIS is an annual, nationally representative, household survey of the noninstitutionalized U.S. civilian population.** In 2021, a total of 29,482 adults were surveyed^††^ (response rate = 50.9%) (*5*). Data were weighted to provide nationally representative estimates, adjusting for differences in selection probability and nonresponse. Use of five tobacco products was assessed: cigarettes, cigars (cigars, cigarillos, or filtered little cigars), pipes (regular pipes, water pipes, or hookahs), e-cigarettes, and smokeless tobacco (chewing tobacco, snuff, dip, snus, or dissolvable tobacco). In this report, tobacco use refers to commercial tobacco products and not to tobacco used for medicinal and spiritual purposes by some American Indian communities. Current cigarette smoking was defined as having ever smoked 100 or more cigarettes within one’s lifetime and smoking every day or some days at the time of survey. Current use of all other tobacco products was defined as use of the product every day or some days at the time of survey. Prevalence estimates for current use of any tobacco product, any combustible tobacco product, two or more tobacco products, and quit ratios^§§^ were calculated; for 2021, estimates were calculated overall and by sex, age group, race and ethnicity, U.S. Census Bureau region,^¶¶^ rural/urban designation,*** educational attainment (among adults aged ≥25 years), marital status, income-to-poverty ratio,^†††^ sexual orientation,^§§§^ health insurance coverage,^¶¶¶^ disability,**** and presence of serious psychological distress.^††††^

The top five combinations of tobacco products reported among adults who reported use of two or more tobacco prod­ucts were determined. Previous year changes in tobacco product use prevalences were calculated. Changes in cigarette smoking during 2019–2021 by diagnosed depression^§§§§^ overall and among racial and ethnic groups were also calculated. SAS-Callable SUDAAN software (version 11.0.3; Research Triangle Institute) was used to conduct all analyses. Chi-square tests were used to test for statistical significance. P-values <0.05 were considered statistically significant. This activity was reviewed by CDC and was conducted consistent with applicable federal law and CDC policy.^¶¶¶¶^

Among U.S. adults in 2021, 18.7% (an estimated 46.0 million) currently used any tobacco product, 14.5% (35.6 million) used any combustible tobacco product, and 3.4% (8.3 million) used two or more tobacco products. Nearly two thirds (66.5%) of adults who ever smoked cigarettes reported having quit (Table). Cigarettes were the most used tobacco product (11.5%; 28.3 million). Prevalence of use and estimated number of users of other tobacco products were as follows: e-cigarettes (4.5%; 11.1 million), cigars (3.5%; 8.6 million), smokeless tobacco (2.1%; 5.2 million), and pipes (0.9%; 2.3 million). Among persons who reported current tobacco product use, 77.5% used combustible tobacco products, and 18.1% used two or more tobacco products. During 2020–2021, a decrease in cigarette smoking prevalence from 12.5% to 11.5% was observed (p<0.01); however, the prevalence of e-cigarette use increased from 3.7% to 4.5% (p<0.01). No statistically significant changes in use prevalences were observed for other tobacco products.

**TABLE:** Characteristics of adults aged ≥18 years who reported tobacco product use “every day” or “some days,” by tobacco product and quit ratios — National Health Interview Survey, United States, 2021

Characteristic	Tobacco product use,* % (95% CI)^†^								Quit ratio^§§§^
	Any tobacco product^§^	Combustible tobacco product^¶^	Cigarettes**	Cigars^††^	Pipes^§§^	E-cigarettes^¶¶^	Smokeless tobacco products***	Two or more tobacco products^†††^	
**Overall**	**18.7 (18.1–19.4)**	**14.5 (14.0–15.1)**	**11.5 (11.1–12.0)**	**3.5 (3.2–3.8)**	**0.9 (0.8–1.1)**	**4.5 (4.2–4.9)**	**2.1 (1.9–2.4)**	**3.4 (3.1–3.7)**	**66.5 (65.4–67.7)**
**Sex**									
Men	24.1 (23.2–25.1)	18.2 (17.4–19.0)	13.1 (12.4–13.9)	6.2 (5.7–6.7)	1.2 (1.0–1.5)	5.1 (4.7–5.6)	4.2 (3.7–4.6)	4.8 (4.4–5.3)	67.4 (65.9–69.0)
Women	13.6 (13.0–14.3)	11.1 (10.4–11.7)	10.1 (9.5–10.7)	1.0 (0.8–1.2)	0.7 (0.5–0.9)	4.0 (3.6–4.4)	0.2 (0.2–0.3)	2.1 (1.8–2.4)	65.4 (63.8–66.9)
**Age group, yrs**									
18–24	17.0 (15.1–19.1)	8.5 (7.1–10.0)	5.3 (4.3–6.6)	3.1 (2.2–4.2)	1.5 (1.0–2.3)	11.0 (9.4–12.7)	1.4 (0.8–2.2)	4.3 (3.3–5.5)	50.3 (42.0–58.6)
25–44	22.1 (21.0–23.2)	16.8 (15.9–17.9)	12.6 (11.8–13.5)	4.9 (4.4–5.5)	1.6 (1.3–1.9)	6.5 (5.9–7.1)	2.5 (2.1–2.9)	5.1 (4.5–5.7)	59.0 (56.7–61.2)
45–64	21.1 (20.0–22.2)	17.5 (16.5–18.5)	14.9 (14.0–15.9)	3.5 (3.0–3.9)	0.4 (0.3–0.6)	2.7 (2.3–3.1)	2.8 (2.4–3.3)	2.9 (2.5–3.3)	62.1 (60.4–63.9)
≥65	11.0 (10.3–11.8)	9.8 (9.0–10.6)	8.3 (7.6–9.0)	1.7 (1.4–2.0)	0.4 (0.3–0.5)	0.9 (0.7–1.1)	0.9 (0.7–1.2)	1.1 (0.8–1.4)	81.9 (80.5–83.3)
**Race and ethnicity** ^¶¶¶^									
AI/AN, non-Hispanic	—****	—****	—****	—****	—****	—****	—****	—****	—****
Asian, non-Hispanic	8.6 (7.0–10.5)	7.0 (5.5–8.8)	5.4 (4.1–6.9)	1.2 (0.7–1.9)	0.9 (0.5–1.6)	2.9 (2.0–4.0)	0.3 (0.1–0.7)	1.8 (1.1–2.8)	70.1 (63.6–76.2)
Black or African American, non-Hispanic	18.1 (16.4–20.0)	16.4 (14.7–18.2)	11.7 (10.3–13.2)	5.1 (4.0–6.3)	2.0 (1.4–2.8)	2.4 (1.8–3.2)	0.9 (0.5–1.4)	3.4 (2.6–4.3)	53.7 (49.5–57.9)
White, non-Hispanic	21.2 (20.4–22.0)	15.9 (15.2–16.6)	12.9 (12.3–13.6)	3.7 (3.4–4.0)	0.8 (0.6–0.9)	5.2 (4.8–5.7)	2.9 (2.6–3.2)	3.8 (3.4–4.1)	67.9 (66.6–69.2)
Hispanic	12.4 (11.2–13.6)	9.9 (8.8–11.0)	7.7 (6.8–8.7)	2.5 (1.9–3.1)	0.9 (0.6–1.2)	3.3 (2.8–4.0)	0.8 (0.5–1.2)	2.2 (1.7–2.7)	67.7 (64.4–70.8)
Other, non-Hispanic	25.6 (21.2–30.4)	18.0 (14.2–22.3)	14.9 (11.4–19.0)	5.2 (2.8–8.6)	1.1 (0.3–2.5)	8.9 (6.2–12.4)	1.2 (0.5–2.4)	5.1 (2.7–8.6)	61.5 (53.5–69.2)
**U.S. Census Bureau region^††††^**									
Northeast	16.2 (14.6–17.8)	13.5 (12.1–15.0)	10.4 (9.2–11.7)	3.5 (2.8–4.4)	0.8 (0.5–1.1)	3.2 (2.6–3.9)	1.2 (0.8–1.6)	2.5 (1.8–3.3)	69.1 (66.0–72.1)
Midwest	22.1 (20.7–23.7)	17.2 (16.0–18.5)	14.0 (12.9–15.2)	4.0 (3.4–4.6)	0.8 (0.5–1.1)	4.6 (3.9–5.4)	3.2 (2.7–3.9)	3.8 (3.2–4.4)	64.0 (61.8–66.1)
South	19.7 (18.6–20.8)	15.4 (14.4–16.3)	12.4 (11.6–13.2)	3.7 (3.3–4.2)	1.1 (0.8–1.4)	4.7 (4.2–5.3)	2.3 (1.9–2.7)	3.8 (3.4–4.3)	64.6 (62.7–66.4)
West	16.0 (14.9–17.1)	11.5 (10.6–12.5)	8.9 (8.0–9.8)	2.8 (2.4–3.3)	1.0 (0.8–1.4)	5.2 (4.5–5.9)	1.6 (1.3–2.0)	3.0 (2.6–3.5)	70.9 (68.5–73.2)
**Urbanization level^§§§§^**									
Urban	17.5 (16.9–18.2)	13.6 (13.1–14.2)	10.5 (10.0–11.1)	3.5 (3.2–3.8)	0.9 (0.8–1.1)	4.4 (4.1–4.8)	1.8 (1.6–2.0)	3.1 (2.8–3.4)	68.1 (66.8–69.4)
Rural	26.2 (24.4–28.1)	20.1 (18.5–21.7)	18.0 (16.5–19.6)	3.5 (2.7–4.4)	0.9 (0.6–1.4)	5.3 (4.4–6.3)	4.5 (3.8–5.3)	5.1 (4.3–6.0)	58.9 (56.5–61.3)
**Educational attainment, adults aged ≥25 yrs**									
0–12 yrs, no diploma	23.6 (21.5–25.8)	21.2 (19.2–23.3)	20.1 (18.2–22.1)	2.8 (2.0–3.7)	0.4 (0.2–0.8)	3.0 (2.2–4.1)	1.7 (1.2–2.3)	3.7 (2.8–4.7)	54.5 (50.9–58.1)
GED	39.0 (34.2–44.1)	33.6 (28.9–38.4)	30.7 (26.2–35.5)	5.2 (3.2–8.0)	0.9 (0.3–2.2)	6.4 (4.4–9.0)	5.4 (3.5–7.9)	8.3 (6.0–11.3)	52.0 (46.2–57.8)
High school diploma	24.4 (23.1–25.8)	19.7 (18.5–21.0)	17.1 (15.9–18.2)	3.4 (2.9–4.1)	1.0 (0.7–1.4)	4.7 (4.0–5.4)	3.1 (2.6–3.7)	4.3 (3.7–5.0)	62.1 (60.0–64.2)
Some college, no degree	23.3 (21.7–25.0)	19.2 (17.6–20.8)	16.1 (14.7–17.6)	4.2 (3.4–5.2)	1.1 (0.7–1.7)	5.0 (4.2–5.9)	2.3 (1.7–2.9)	4.5 (3.7–5.5)	64.2 (61.5–66.7)
Associate degree (academic, technical, or vocational)	21.5 (19.9–23.2)	16.8 (15.3–18.3)	13.7 (12.3–15.1)	4.0 (3.2–4.9)	0.9 (0.6–1.3)	4.8 (3.9–5.8)	3.0 (2.3–3.8)	4.1 (3.3–5.0)	67.4 (64.6–70.2)
Bachelor’s degree	12.0 (11.1–12.9)	9.1 (8.3–9.9)	5.3 (4.7–5.9)	3.8 (3.3–4.4)	0.8 (0.6–1.1)	2.6 (2.1–3.1)	1.6 (1.3–2.0)	1.8 (1.4–2.1)	80.3 (78.2–82.4)
Graduate degree (master's, doctoral, or professional)	8.6 (7.6–9.6)	6.5 (5.6–7.4)	3.2 (2.7–3.9)	2.9 (2.4–3.6)	0.8 (0.6–1.2)	2.0 (1.5–2.5)	0.9 (0.6–1.3)	1.2 (0.8–1.6)	86.3 (83.7–88.6)
**Marital status**									
Married or living with partner	17.5 (16.7–18.2)	13.4 (12.7–14.1)	10.4 (9.8–11.1)	3.4 (3.1–3.8)	0.7 (0.6–0.9)	3.7 (3.3–4.1)	2.3 (2.0–2.6)	2.8 (2.4–3.1)	71.1 (69.7–72.5)
Divorced, separated, or widowed	21.3 (20.1–22.5)	18.7 (17.6–19.8)	16.8 (15.7–17.9)	2.9 (2.4–3.4)	0.6 (0.4–0.9)	3.5 (2.9–4.1)	2.0 (1.7–2.5)	3.7 (3.2–4.3)	64.1 (62.1–66.1)
Single, never married, or not living with partner	20.1 (18.8–21.5)	14.5 (13.4–15.7)	10.9 (9.9–11.8)	4.2 (3.6–4.9)	1.8 (1.4–2.2)	7.5 (6.6–8.4)	1.7 (1.4–2.1)	4.8 (4.2–5.5)	51.6 (48.7–54.5)
**Income to poverty ratio (income level)^¶¶¶¶^**									
0–1.99 (low)	24.7 (23.4–26.1)	20.6 (19.4–21.8)	18.3 (17.2–19.5)	3.4 (2.9–4.0)	1.2 (0.9–1.6)	5.9 (5.2–6.6)	1.9 (1.6–2.4)	5.0 (4.5–5.7)	46.1 (42.4–9.8)
2.00–3.99 (middle)	18.9 (17.9–20.0)	14.8 (13.9–15.8)	12.3 (11.5–13.3)	3.1 (2.6–3.6)	0.9 (0.6–1.1)	4.6 (4.0–5.2)	2.1 (1.7–2.5)	3.5 (3.0–4.1)	56.9 (54.4–9.5)
≥4.00 (high)	14.8 (14.0–15.6)	10.5 (9.8–11.1)	6.7 (6.2–7.3)	3.8 (3.4–4.3)	0.8 (0.6–1.0)	3.6 (3.2–4.1)	2.3 (2.0–2.7)	2.3 (2.0–2.6)	72.5 (71.2–73.8)
**Sexual orientation**									
Heterosexual or straight	18.4 (17.7–19.1)	14.3 (13.8–14.9)	11.4 (10.9–11.9)	3.5 (3.2–3.8)	0.9 (0.7–1.0)	4.1 (3.8–4.5)	2.2 (2.0–2.5)	3.2 (3.0–3.5)	66.9 (65.7–68.1)
Lesbian, gay, or bisexual	27.4 (24.1–30.9)	18.8 (16.1–21.6)	15.3 (12.9–17.9)	4.1 (2.8–5.8)	2.3 (1.3–3.7)	13.2 (10.6–16.1)	1.2 (0.6–2.1)	7.2 (5.4–9.3)	61.3 (55.9–66.5)
Health insurance coverage*****									
Private	16.2 (15.5–17.0)	11.7 (11.1–12.3)	8.6 (8.1–9.1)	3.5 (3.1–3.8)	0.7 (0.6–0.9)	4.1 (3.8–4.5)	2.3 (2.0–2.6)	2.6 (2.3–2.9)	72.4 (71.0–73.8)
Medicaid	28.1 (26.1–30.1)	24.1 (22.2–26.0)	21.5 (19.9–23.3)	3.7 (3.0–4.6)	1.6 (1.1–2.2)	6.7 (5.6–8.0)	1.8 (1.2–2.5)	5.9 (4.9–7.0)	46.7 (43.6–49.8)
Medicare only (age ≥65 yrs)	10.7 (9.4–12.0)	9.8 (8.6–11.0)	8.4 (7.3–9.6)	1.4 (1.0–1.9)	0.4 (0.2–0.8)	0.9 (0.5–1.3)	0.8 (0.5–1.2)	1.2 (0.7–1.8)	81.1 (78.6–83.4)
Other public insurance	21.6 (19.3–24.1)	17.2 (15.1–19.5)	13.9 (12.0–16.0)	4.4 (3.2–6.0)	1.1 (0.6–1.9)	4.1 (3.0–5.5)	2.3 (1.5–3.3)	3.9 (2.8–5.4)	71.1 (67.3–74.7)
None	28.4 (26.1–30.9)	23.5 (21.5–25.6)	20.0 (18.1–22.1)	5.0 (4.0–6.1)	2.0 (1.3–2.9)	7.2 (6.0–8.7)	2.5 (1.9–3.3)	7.1 (5.9–8.5)	43.8 (40.1–47.6)
**Disability^†††††^**									
Yes	24.2 (22.3–26.2)	20.4 (18.7–22.2)	18.5 (16.8–20.2)	3.4 (2.6–4.3)	1.1 (0.7–1.6)	4.7 (3.7–5.9)	1.7 (1.2–2.4)	4.2 (3.4–5.3)	64.0 (61.2–66.8)
No	18.2 (17.5–18.8)	13.9 (13.4–14.5)	10.9 (10.4–11.4)	3.5 (3.2–3.8)	0.9 (0.8–1.1)	4.5 (4.2–4.9)	2.2 (1.9–2.4)	3.3 (3.0–3.6)	66.9 (65.7–68.1)
**Serious psychological distress^§§§§§^**									
Yes	37.6 (34.0–41.3)	32.1 (28.6–35.7)	28.1 (24.8–31.6)	6.5 (4.7–8.6)	2.7 (1.6–4.4)	10.4 (8.2–12.9)	1.7 (1.0–2.9)	9.8 (7.7–12.2)	45.3 (40.2–50.5)
No	18.0 (17.4–18.7)	13.9 (13.3–14.4)	10.9 (10.4–11.4)	3.4 (3.1–3.7)	0.9 (0.7–1.0)	4.3 (4.0–4.7)	2.2 (1.9–2.4)	3.1 (2.9–3.4)	67.7 (66.6–68.9)

**Abbreviations**: AI/AN = American Indian or Alaska Native; CHIP = Children’s Health Insurance Program; e-cigarettes = electronic cigarettes; GED = general educational development certificate; NCHS = National Center for Health Statistics; NHIS = National Health Interview Survey.

* Smoking and tobacco product use refers to use of commercial tobacco products and not to tobacco used for medicinal and spiritual purposes by some American Indian communities.

^†^ 95% Korn-Graubard CIs. NCHS data presentation standards. https://www.cdc.gov/nchs/data/series/sr_02/sr02_175.pdf

^§^ Any tobacco use was defined as use either “every day” or “some days” of at least one tobacco product. For cigarettes, users were defined as adults who reported use either “every day” or “some days” and had smoked ≥100 cigarettes during their lifetime.

^¶^ Any combustible tobacco use was defined as use either “every day” or “some days” of at least one combustible tobacco product: cigarettes; cigars, cigarillos, filtered little cigars; pipes, water pipes, or hookah. For cigarettes, users were defined as adults who reported use either “every day” or “some days” and had smoked 100 or more cigarettes during their lifetime.

** Current cigarette smoking was defined as smoking 100 or more cigarettes during a person’s lifetime and now smoking cigarettes “every day” or “some days.”

^††^ Current cigar smoking was defined as smoking cigars, cigarillos, or little filtered cigars at least once during a person’s lifetime and now smoking at least one of these products “every day” or “some days.”

^§§^ Current pipe smoking was defined as smoking tobacco in a regular pipe, water pipe, or hookah at least once during a person’s lifetime and now smoking at least one of these products “every day” or “some days.”

^¶¶^ Current e-cigarette use was defined as using e-cigarettes at least once during a person’s lifetime and now using e-cigarettes “every day” or “some days.”

*** Current smokeless tobacco product use was defined as using chewing tobacco, snuff, dip, snus, or dissolvable tobacco at least once during a person’s lifetime and now using at least one of these products “every day” or “some days.”

^†††^ Current multiple tobacco product use was defined as use “every day” or “some days” for two or more of the following tobacco products: cigarettes (100 or more cigarettes during a person’s lifetime); cigars, cigarillos, filtered little cigars; pipes, water pipes, or hookah; e-cigarettes; or smokeless tobacco products.

^§§§^ Quit ratio is the percentage of persons who ever smoked (100 or more cigarettes during their lifetime) who have quit smoking.

^¶¶¶^ Hispanic persons could be of any race. All other groups were non-Hispanic. The following four non-Hispanic single-race categories were available for sampled adults in the 2021 NHIS public use files: 1) AI/AN; 2) Asian; 3) Black or African American; and 4), White. The “non-Hispanic, other” category includes those adults who were categorized as “non-Hispanic AI/AN and any other group” or “other single and multiple races.”

**** Based on NCHS data presentation standards, estimates were statistically unreliable (https://www.cdc.gov/nchs/data/series/sr_02/sr02_175.pdf). SAS MACRO used to suppress criteria check. https://www.sas.com/content/dam/SAS/support/en/sas-global-forum-proceedings/2019/3659-2019.pdf

^††††^ https://www2.census.gov/geo/pdfs/maps-data/maps/reference/us_regdiv.pdf

^§§§§^ Urbanization level (termed metropolitan statistical area in a previous report) is based on the 2013 NCHS Urban-Rural Classification Scheme for Counties (https://www.cdc.gov/nchs/data/series/sr_02/sr02_166.pdf). Note that the original variable for urbanization level in the NHIS public use data files is four levels; for this report, the four-level variable has been reduced to a dichotomous variable.

^¶¶¶¶^ Ratio of family income to poverty threshold for family size based on the imputed family income to poverty threshold variable (29,482).

***** *Private coverage:* adults who had any comprehensive private insurance plan (including health maintenance organizations and preferred provider organizations). *Medicaid:* adults aged <65 years; includes those who did not have private coverage but had Medicaid or other state-sponsored health plans, including CHIP. For adults aged ≥65 years, those who did not have any private coverage but had Medicare and Medicaid or other state-sponsored health plans were categorized as having Medicaid. *Medicare only*: adults aged ≥65 years who only had Medicare coverage. *Other coverage:* adults who did not have private insurance, Medicaid, or other public coverages, but who had any type of military coverage, coverage from other government programs, or Medicare (adults aged <65 years). *Uninsured*: adults who did not indicate at the time of interview that they were covered under private health insurance, Medicare, Medicaid, CHIP, a state-sponsored health plan, other government programs, or military coverage. Insurance coverage is “as of time of survey.”

^†††††^ Disability was defined based on self-reported presence of selected limitations including vision, hearing, mobility, remembering or concentrating, self-care, and communication. Respondents who indicated “A lot of difficulty” or “Cannot do at all/unable to do” to at least one of the following six questions: “Do you have difficulty seeing, even when wearing glasses?,” “Do you have difficulty hearing, even when using a hearing aid?,” “Do you have any difficulty walking or climbing steps?,” “Using your usual language, do you have difficulty communicating, for example, understanding or being understood?,” “Do you have difficulty remembering or concentrating?,” or “Do you have difficulty with self-care, such as washing all over or dressing?” were coded as living with a disability; those who responded “no difficulty” or “some difficulty” to all six questions were coded as no disability. These six questions are based on the short set of questions recommended by the Washington Group on Disability Statistics. https://www.cdc.gov/nchs/washington_group/index.htm

^§§§§§^ Serious psychological distress was assessed using a set of questions from the six-item Kessler (K6) scale (https://pubmed.ncbi.nlm.nih.gov/22351472/) that asks sampled adults to assess the frequency of six characteristics (sadness, nervousness, restlessness, hopelessness, feeling that everything was an effort, and worthlessness) experienced within the previous 30 days. https://ftp.cdc.gov/pub/Health_Statistics/NCHS/Dataset_Documentation/NHIS/2021/srvydesc-508.pdf

Among adults reporting the use of two or more tobacco products, the most frequently reported combination was cigarettes and e-cigarettes (31.4%), followed by cigarettes and cigars (21.0%), cigarettes and smokeless tobacco (7.9%), e-cigarettes and cigars (7.0%), and cigarettes and pipes (3.7%); in addition, 29.0% of adults who currently used two or more tobacco products reported use of some other combination, including 14.0% who reported use of three or more products (Figure 1).

**FIGURE 1:**
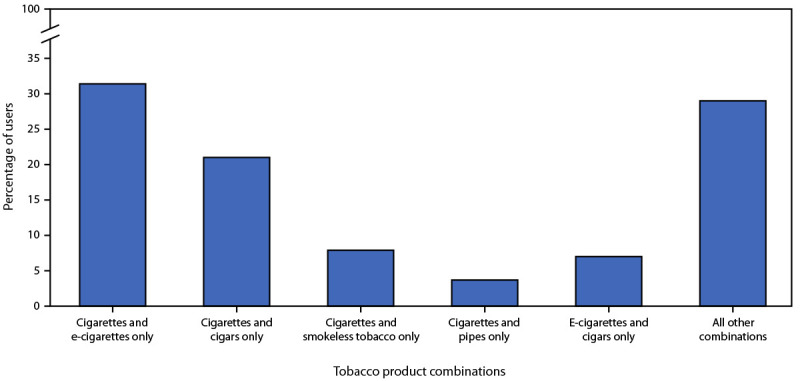
Percentage of persons aged ≥18 years who reported use of two or more tobacco products, by the top five product combinations — United States, 2021*^,^^†^^,^^§^ **Abbreviation:** e-cigarettes = electronic cigarettes. * Smoking and tobacco product use refers to use of commercial tobacco products and not to tobacco used for medicinal and spiritual purposes by some American Indian communities. ^†^ Current smokeless tobacco product use was defined as using chewing tobacco, snuff, dip, snus, or dissolvable tobacco at least once during a person’s lifetime and now using at least one of these products “every day” or “some days.” ^§^ “All other combinations” refers to use of other combinations of two or more products.

During 2019–2021, the prevalence of cigarette smoking among adults who were ever told by a health care provider that they had depression (22.9%, 20.5%, and 19.4% in 2019, 2020, and 2021, respectively) was higher than that among those who had never been told that they had depression (12.3%, 10.9%, and 9.9% in 2019, 2020, and 2021, respectively) (Figure 2). Regardless of diagnosed depression status, statistically significant declines in the prevalence of cigarette smoking during 2019–2021 were noted overall, among non-Hispanic Black or African American (Black); White; and Hispanic adults. During 2019–2021, among adults who ever had diagnosed depression, the prevalence of cigarette smoking was highest among non-Hispanic adults of other races in 2019 and 2020, and highest among Black adults in 2021. Among adults who never had diagnosed depression, cigarette smoking prevalence was similar among Black and White adults and was highest among non-Hispanic American Indian or Alaska Native (AI/AN) adults (in 2020), and non-Hispanic adults of other races (2019 and 2021).

**FIGURE 2:**
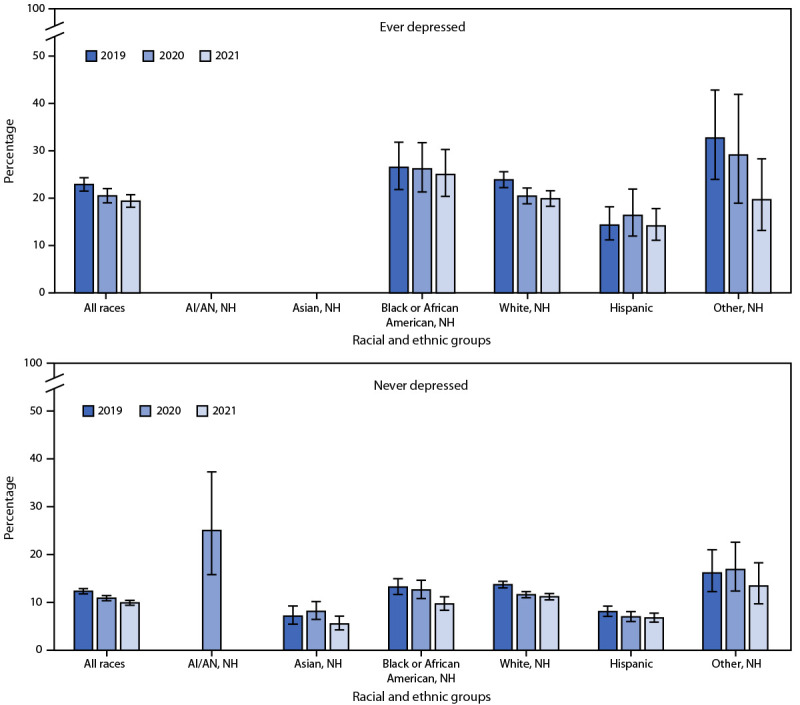
Prevalence* of cigarette smoking^†^ among persons aged ≥18 years, by race and ethnicity and depression diagnosis history^§^ — United States, 2019–2021^¶^^,^**^,^^††^^,^^§§^ **Abbreviations:** AI/AN = American Indian or Alaska Native; NCHS = National Center for Health Statistics; NH = non-Hispanic. * With 95% CIs indicated by error bars. ^†^ Smoking and tobacco product use refers to use of commercial tobacco products and not to tobacco used for medicinal and spiritual purposes by some American Indian communities. ^§^ Depression was defined using the question, “Have you ever been told by a doctor or other health professional that you had … any type of depression?” Those responding “Yes” were classified as having ever had depression in their lifetime. Those responding “No” were classified as never having had depression in their lifetime. ^¶^ Among adults who ever had diagnosed depression, statistically significant (p<0.05) trends during 2019–2021 were observed for the following groups: all races; Black or African American, NH; White, NH; and Hispanic adults. **Among adults who never had diagnosed depression, statistically significant (p<0.05) trends during 2019–2021 were observed for the following groups: all races; Black or African American, NH; White, NH; and Hispanic adults. ^††^ Among adults who ever had diagnosed depression, estimates were statistically unreliable for AI/AN, NH; and Asian, NH adults during 2019–2021 and are not presented based on NCHS data presentation standards (https://www.cdc.gov/nchs/data/series/sr_02/sr02_175.pdf). SAS MACRO used to suppress criteria check. https://support.sas.com/resources/papers/proceedings19/3659-2019.pdf ^§§^ Among adults who never had diagnosed depression, estimates were statistically unreliable for AI/AN, NH adults in 2019 and 2021 and are not presented based on NCHS data presentation standards (https://www.cdc.gov/nchs/data/series/sr_02/sr02_175.pdf). SAS MACRO used to suppress criteria check. https://support.sas.com/resources/papers/proceedings19/3659-2019.pdf

In 2021, the prevalence of any current tobacco product use was higher among men (24.1%) than among women (13.6%) (Table). It was also higher among persons aged 25–44 years (22.1%), 45–64 years (21.1%), and 18–24 years (17.0%) than among those aged ≥65 years (11.0%), and higher among non-Hispanic adults of other races (25.6%) or White (21.2%) adults than among Black (18.1%), Hispanic (12.4%), or non-Hispanic Asian (8.6%) adults. Prevalence was higher among adults living in the Midwest (22.1%) or the South (19.7%) U.S. Census Bureau regions than among those living in the Northeast (16.2%) or West (16.0%), as well as higher among those living in rural areas (26.2%) compared with those in urban areas (17.5%). A larger percentage of adults with a GED certificate (39.0%) reported current tobacco product use than did persons with other levels of education (range = 8.6%–24.4%). In addition, use prevalence was higher among persons who were divorced, separated, or widowed (21.3%) or single, never married, or not living with a partner (20.1%) than among those who were married or living with a partner (17.5%). Other sociodemographic groups with higher prevalences of current tobacco use included persons with low income (24.7%) compared with persons with middle (18.9%) or high income (14.8%), and LGB adults (27.4%) compared with heterosexual/straight adults (18.4%). Higher prevalences of tobacco use were reported by persons who were uninsured (28.4%) or insured by Medicaid (28.1%) than by persons who had some other public insurance (21.6%), private insurance (16.2%), or Medicare only (10.7%); by those with a disability (24.2%) compared with those who did not have a disability (18.2%); and by those who reported serious psychological distress (37.6%) compared with those who did not report serious psychological distress (18.0%).

## Discussion

In 2021, approximately one in nine (11.5%) U.S. adults aged ≥18 years currently smoked cigarettes. Although this finding represents the lowest smoking prevalence recorded since 1965 (*1*), nearly one in five adults continue to use tobacco products. Cigarettes and other combustible tobacco products constitute the largest proportion of overall tobacco product use and are the foremost cause of tobacco-related morbidity and mortality in the United States (*1*). Consistent with the declines during recent decades, the prevalence of cigarette smoking decreased during 2020–2021. Comprehensive tobacco control strategies (e.g., smoke-free laws, media campaigns such as Tips from Former Smokers, and price increases) at the national, state, and local levels likely contributed to the lower cigarette smoking prevalence (*3*,*4*).

Nearly one in five adults who currently used tobacco products used two or more products, with nearly one third of these persons (31.4%) reporting use of both cigarettes and e-cigarettes. Further, the prevalence of e-cigarette use increased during 2020–2021. In addition to continued surveillance of tobacco product use, it is equally important to monitor use of combinations of tobacco products to characterize multiproduct use trends.

Adults identifying as LGB, living in rural areas, or who are uninsured or have Medicaid insurance continue to report high prevalences of tobacco product use (*6*). Further, smoking prevalence is higher among adults who ever had diagnosed depression than among those who never had diagnosed depression, although, consistent with previous studies, prevalence declined from 2019 to 2021 (*1*,*6*,*7*). Racial and ethnic differences in tobacco product use are more pronounced among adults who ever had diagnosed depression than among those who never had diagnosed depression. Racial and ethnic differences in smoking prevalence might be related to a combination of lived experiences, such as discrimination and frequent tobacco product advertising exposure: disproportionate tobacco product advertising in low-income neighborhoods or areas with high proportions of Black or AI/AN persons has been documented (*1*,*8*,*9*). Racial and ethnic differences in smoking prevalence by diagnosed depression might also be partially attributed to differences in access to mental health care and tobacco cessation services (*8*,*9*).

The findings in this report are subject to at least five limitations. First, because NHIS is limited to noninstitutionalized U.S. civilian populations, results are not generalizable to institutionalized populations or persons in the military. Second, responses to questions were self-reported and were not validated by biochemical testing; however, self-reported smoking status has been determined to correlate with serum cotinine levels (*10*). Third, 2021 tobacco product estimates for AI/AN populations were not statistically reliable***** and therefore are not presented. Fourth, changes in the 2020 and 2021 NHIS survey administration from in-person to primarily telephone-based (because of the COVID-19 pandemic) might affect 2020 and 2021 estimates^†††††^ (*5*). Finally, these data are cross-sectional, and trends in tobacco product use changes among individual persons cannot be assessed.

In 2021, the 11.5% prevalence of current cigarette smoking was less than the 12% end-of-decade goal set by Healthy People 2020^§§§§§^. Although cigarette smoking decreased over the past year, e-cigarette use increased, from 3.7% to 4.5%, largely driven by higher prevalence in use among persons aged 18–24 years. Further, declines in cigarette smoking among populations with diagnosed depression represent important successes in tobacco control (*7*). However, disparities in tobacco use remain. Continued implementation of evidence-based strategies, such as increasing the unit price of tobacco products, enforcing comprehensive smoke-free policies, and conducting linguistically and culturally appropriate educational campaigns, combined with FDA regulation of tobacco products and innovative strategies, will support activities and programs to reduce tobacco use and tobacco-related disparities (*3*,*4*).

**Summary**

**What is already known about this topic?**

Tobacco product use remains the leading cause of preventable disease and death in the United States.

**What is added by this report?**

In 2021, approximately 18.7% (46 million) of U.S. adults currently used any tobacco product. Cigarettes were the most frequently reported tobacco product (11.5%), followed by e-cigarettes (4.5%). During 2020–2021, the prevalence of cigarette smoking decreased, and prevalence of e-cigarette use increased.

**What are the implications for public health practice?**

Continued use of evidence-based comprehensive tobacco control strategies, including linguistically and culturally competent educational campaigns and innovative strategies, combined with Food and Drug Administration regulation of tobacco products will support activities and programs to further reduce tobacco-related disease, death, and disparities.

## Footnotes

* The use of regular pipe, water pipe, or hookah was assessed together using a single question. Interviewers could read the following sentences if necessary: “A hookah is a type of water pipe. It is sometimes called a ‘narghile’ (NAR-ge-lee) pipe. Do not include electronic hookahs or e-hookahs”; “Do not include electronic pipes or e-pipes”; “Do not include pipes filled with substances other than tobacco.”

† Categories are not mutually exclusive.

§ Current use of two or more tobacco products was defined as use either “every day” or “some days” of at least two or more of the following tobacco products: cigarettes (100 or more cigarettes during a person’s lifetime); cigars, cigarillos, or filtered little cigars; pipes, water pipes, or hookahs; electronic cigarettes; or smokeless tobacco products.

¶ Hispanic persons could be of any race. All other groups were non-Hispanic. The following four non-Hispanic single-race categories were available for sampled adults in the 2021 NHIS public use files: 1) American Indian or Alaska Native (AI/AN); 2) Asian; 3) Black or African American; and 4), White. The “non-Hispanic, other” category includes those adults who were categorized as “non-Hispanic AI/AN and any other group” or “other single and multiple races.”

**
https://ftp.cdc.gov/pub/Health_Statistics/NCHS/Dataset_Documentation/NHIS/2021/srvydesc-508.pdf

†† Because of the COVID-19 pandemic, typical data collection procedures were disrupted. During January–April 2021, contact with household members was attempted first via telephone, with subsequent personal visits allowed (a continuation of procedures from the latter part of 2020). Beginning in May 2021, interviewers returned to regular procedures, whereby first contact with household members was attempted in person, with follow-up allowed by telephone. Allowed personal visits varied by regional office, based on local COVID-19 rates. Interviewers were required to wear masks, practice social distancing, and be fully vaccinated if in-home interviews were conducted. During 2021, 62.8% of interviews were conducted at least partially by telephone compared with 34.3% of interviews in 2019.

§§ Quit ratio is the percentage of persons who have ever smoked (100 or more cigarettes during their lifetime) and have quit smoking.

¶¶ https://www2.census.gov/geo/pdfs/maps-data/maps/reference/us_regdiv.pdf

*** Rural/urban designation (urbanization level), termed metropolitan statistical area in a previous report, is based on the 2013 National Center for Health Statistics (NCHS) Urban-Rural Classification Scheme for Counties (https://www.cdc.gov/nchs/data/series/sr_02/sr02_166.pdf). The original variable for urbanization level in the NHIS public use data files is four levels; for this report, the four-level variable is reduced to a dichotomous variable.

††† Ratio of family income to poverty threshold for family size variable based on the imputed family income to poverty threshold variable (29,482).

§§§ Sexual orientation was determined using the question, “Which of the following best represents how you think of yourself?” Response options included “gay,” “straight, that is, not gay,” “bisexual,” “something else,” and “I don’t know the answer” among male respondents, and “lesbian or gay,” “straight, that is, not lesbian or gay,” “bisexual,” “something else,” and “I don’t know the answer” among female respondents. Respondents were considered to be LGB if they responded “gay,” “lesbian or gay,” or “bisexual.”

¶¶¶ *Private coverage*: adults who had any comprehensive private insurance plan (including health maintenance organizations and preferred provider organizations). *Medicaid*: adults aged <65 years; includes those who did not have private coverage, but had Medicaid or other state-sponsored health plans, including the Children’s Health Insurance Program (CHIP). For adults aged ≥65 years, those who did not have any private coverage but had Medicare and Medicaid or other state-sponsored health plans were categorized as having Medicaid. *Medicare only*: adults aged ≥65 years who only had Medicare coverage. *Other coverage*: adults who did not have private insurance, Medicaid, or other public coverages, but who had any type of military coverage, coverage from other government programs, or Medicare (adults aged <65 years). *Uninsured*: adults who did not indicate at the time of interview that they were covered under private health insurance, Medicare, Medicaid, CHIP, a state-sponsored health plan, other government programs, or military coverage. Insurance coverage is “as of time of survey.”

**** Disability was defined based on self-reported presence of selected limitations including vision, hearing, mobility, remembering or concentrating, self-care, and communication. Respondents who indicated “A lot of difficulty” or “Cannot do at all/unable to do” to at least one of the following six questions: “Do you have difficulty seeing, even when wearing glasses?,” “Do you have difficulty hearing, even when using a hearing aid?,” “Do you have any difficulty walking or climbing steps?,” “Using your usual language, do you have difficulty communicating, for example, understanding or being understood?,” “Do you have difficulty remembering or concentrating?,” or “Do you have difficulty with self-care, such as washing all over or dressing?” were coded as living with a disability; those who responded “no difficulty” or “some difficulty” to all six questions were coded as no disability. These six questions are based on the short set of questions recommended by the Washington Group on Disability Statistics. https://www.cdc.gov/nchs/washington_group/index.htm

†††† Serious psychological distress was assessed using a set of questions from the six-item Kessler (K6) scale (https://pubmed.ncbi.nlm.nih.gov/22351472/) that asks sampled adults to assess the frequency of six characteristics (sadness, nervousness, restlessness, hopelessness, feeling that everything was an effort, and worthlessness) experienced within the previous 30 days. https://ftp.cdc.gov/pub/Health_Statistics/NCHS/Dataset_Documentation/NHIS/2021/srvydesc-508.pdf

§§§§ Depression was defined using the question, “Have you ever been told by a doctor or other health professional that you had… any type of depression?” Those responding “Yes” were classified as having ever had depression in their lifetime and those responding “No” as never having had depression in their lifetime.

¶¶¶¶ 45 C.F.R. part 46.102(l)(2), 21 C.F.R. part 56; 42 U.S.C. Sect. 241(d); 5 U.S.C. Sect. 552a; 44 U.S.C. Sect. 3501 et seq.

***** Based on NCHS data presentation standards, estimates were statistically unreliable (https://www.cdc.gov/nchs/data/series/sr_02/sr02_175.pdf). SAS MACRO used to suppress criteria check. https://www.sas.com/content/dam/SAS/support/en/sas-global-forum-proceedings/2019/3659-2019.pdf

†††††
https://ftp.cdc.gov/pub/Health_Statistics/NCHS/Dataset_Documentation/NHIS/2020/nonresponse-report-508.pdf

§§§§§
https://wayback.archive-it.org/5774/20220415165927/https://www.healthypeople.gov/2020/topics-objectives/topic/tobacco-use/objectives
